# MPRAnator: a web-based tool for the design of massively parallel reporter assay experiments

**DOI:** 10.1093/bioinformatics/btw584

**Published:** 2016-09-06

**Authors:** Ilias Georgakopoulos-Soares, Naman Jain, Jesse M Gray, Martin Hemberg

**Affiliations:** 1 Department of Computational Genomics, Wellcome Trust Sanger Institute, Wellcome Genome Campus, Hinxton, UK; 2 Department of Life Sciences, Imperial College London, London, UK; 3 Department of Genetics, Harvard Medical School, Boston, MA, USA

## Abstract

**Motivation:**

With the rapid advances in DNA synthesis and sequencing technologies and the continuing decline in the associated costs, high-throughput experiments can be performed to investigate the regulatory role of thousands of oligonucleotide sequences simultaneously. Nevertheless, designing high-throughput reporter assay experiments such as massively parallel reporter assays (MPRAs) and similar methods remains challenging.

**Results:**

We introduce MPRAnator, a set of tools that facilitate rapid design of MPRA experiments. With MPRA Motif design, a set of variables provides fine control of how motifs are placed into sequences, thereby allowing the investigation of the rules that govern transcription factor (TF) occupancy. MPRA single-nucleotide polymorphism design can be used to systematically examine the functional effects of single or combinations of single-nucleotide polymorphisms at regulatory sequences. Finally, the Transmutation tool allows for the design of negative controls by permitting scrambling, reversing, complementing or introducing multiple random mutations in the input sequences or motifs.

**Availability and implementation:**

MPRAnator tool set is implemented in Python, Perl and Javascript and is freely available at www.genomegeek.com and www.sanger.ac.uk/science/tools/mpranator. The source code is available on www.github.com/hemberg-lab/MPRAnator/ under the MIT license. The REST API allows programmatic access to MPRAnator using simple URLs.

**Supplementary information:**

Supplementary data are available at *Bioinformatics* online.

## 1 Introduction

DNA synthesis and sequencing technology is advancing rapidly, allowing for the design of high-throughput experiments, which were previously hindered by technological constraints. In a single massively parallel reporter assay (MPRA) experiment, thousands of oligonucleotides are synthesized on microarrays, each linked to a unique identifier (Melnikov *et al.*, 2012), (Patwardhan *et al.*, 2012). The oligonucleotides are amplified, integrated into plasmids in front of a reporter gene and transfected into cells. By measuring the expression levels of the reporter gene using RNA-seq, the regulatory properties of the corresponding sequences can be quantified [for reviews on MPRAs, see Dailey (2015) and Inoue and Ahituv (2015)].

MPRA experiments have been implemented to study the relative positioning of transcription factor binding sites and their regulatory effects in modulating gene expression (Mogno *et al.*, 2013; Sharon *et al.*, 2012, 2014). They can also be employed to systematically investigate the regulatory effects of single-nucleotide polymorphisms (SNPs), thereby relating information provided from genome-wide association studies at the population level with the exploration of functional effects at the cellular level.

Even though decreasing costs have made MPRA experimental procedure accessible to most labs, widespread adoption of the method is limited by computational challenges. Since each MPRA array can involve tens of thousands of different sequences, it is very hard to manually design MPRA experiments, as there are a plethora of parameters that need to be adjusted. Here we present MPRAnator, a set of tools that allow systematic design of MPRA experiments for the investigation of the effects of SNPs and motifs on regulatory sequences.

## 2 Materials and methods

The overarching aim of MPRAnator is to allow users to systematically design synthetic DNA sequences for high-throughput experiments in an interactive manner. Currently, MPRAnator provides support for four different types of investigations. The MPRA Motif design tool can be used to systematically generate synthetic sequences with single motifs or combinations of motifs placed at preselected positions. The MPRA SNP design tool can be used to examine the regulatory effects of single or combinations of SNPs for every provided sequence. The PWM Seq-Gen tool performs probabilistic realizations of PWMs or generates all the corresponding k-mer motifs exceeding a probability threshold. The Transmutation tool allows for the design of different types of negative controls for MPRA experiments. More details for each tool can be found in the Supplementary Material.

The regulatory effects of both motifs and SNPs can be studied in isolation as well as combinatorially. The MPRAnator tool set is highly flexible allowing for the incorporation of other genomic sequences as sub-components. These include uniquely identifiable barcodes, adapters and restriction sites or other sequence types of interest. Using a drag and drop option, the user can select the ordering of the sub-components into the final experimental design. The generated sequences can be incorporated into different types of vectors, such as viruses or plasmids by introducing the relevant subcomponents into the final sequence product. Therefore, MPRAnator could be used for other types of high-throughput designs besides MPRA experiments. Lastly, the user-friendly nature of MPRAnator will facilitate further adoption of MPRA technology.

## Supplementary Material

Supplementary Data
