# Supplementary material for: MPRAnator: a web-based tool for the design of massively parallel reporter assay experiments
Source: Bioinformatics. 2016 Sep 6;33(1):137–8. doi: 10.1093/bioinformatics/btw584 (PMC5198521; doi:10.1093/bioinformatics/btw584)
Supplement: Supplementary Data [file btw584_supp.docx]

**Supplementary**

**2.1. MPRAnator Motif Design Tool**

The MPRAnator Motif Design Tool requires a minimum of two inputs: i) a set of FASTA scaffold sequences and ii) a list of motifs in FASTA format. It outputs a set of FASTA sequences, where a subset of the nucleotides in the scaffold was substituted for the different motifs that were provided by the user. A set of optional inputs provides the user with fine control of how the motifs are placed. The user can adjust the frequency of motif substitutions into the sequences and use restriction parameters to control the relative distance between motifs substituted in the sequence as well as the range within the sequence at which it is permitted to substitute motifs. (Fig.1) Moreover, the user can select to incorporate uniquely identifiable barcodes to the output sequences, select the barcode size (set to zero to exclude barcodes), select the minimum Levenshtein distance between barcodes and restrict the range of the barcode GC content. The Levenshtein distance corresponds to the minimum distance that is needed to convert one sequence to another, involving deletions, insertions and translocations. Furthermore, the user can select the multiplicity of each sequence to optimize the experiment. If multiple motifs are inputted, sequences will be generated for each motif separately and for the motif combinations. For instance, if three motifs are inputted, output sequences for single, pairs and triplets of motifs will be generated, following the restrictions imposed by the user. On the website all parameters are set to sensible default values. To facilitate integration of the sequence to a vector, restriction sites, adaptor sequences or other sequences of interest can be added. In the output file, the headers of the scaffold sequences contain the corresponding information including the motif sequences, the positions of substitution and any additional subcomponents added to the final product. If any of the restriction sites are identified in any generated oligonucleotide sequence they are reported in the header of the corresponding sequence. Substituted motifs are colour-marked for visualization purposes. Importantly, the tool supports a modular design and the order of the constituent parts can be easily altered using a drag and drop interface. MPRAnator allows for a very high degree of flexibility in the design of the experiment and it is straightforward for the user to explore a wide range of sequence layouts. The flexible layout means that the output sequences are not restricted to MPRA experiments, but they can easily be linked to similar protocols such as BunDLE-seq (Levo *et al.*, 2015).

**2.2 MPRAnator SNP design tool:**

The MPRAnator SNP design tool uses two inputs: i) a set of FASTA scaffold sequences and ii) a list of associated SNPs represented using the variant call file (VCF) format. The VCF format supports the use of up to 12 columns for each locus, but MPRAnator only uses the information found in the first 5 columns. For each sequence in the FASTA file, MPRAnator will substitute the associated SNPs. If more than one SNP is found at a given locus, all combinations will be generated (Fig. 2). Deletions or insertions (up to 10nt) in VCF format are also allowed. Since several methods of oligonucleotide synthesis work best when all oligos have similar lengths, the instances with an insertion must be trimmed while the sequences with a deletion must be expanded. MPRAnator solves this problem by adding adenines to one end of the sequences that are too short, and by trimming one end of the sequences that are too long (Fig.2).

As with the motif design tool, a set of optional inputs can be selected for optimal design of the experiment. The user can select to incorporate uniquely identifiable barcodes to the output sequences, select the barcode size (set to zero to exclude barcodes), minimum Levenshtein distance between barcodes and restrict the range of the barcode GC content. Although the default is to substitute all combinations of SNPs in the input sequences, there is also the option to substitute only a single SNP at a time. To facilitate integration of the sequence to a vector, restriction sites, adaptor sequences or other sequences of interest can be added. The headers of the scaffold sequences contain all the corresponding information, including the SNP names, their position and their sequence as well as any additional subcomponents added into the final product. If any of the restriction sites that have been added to the scaffold sequences are identified in any generated oligonucleotide sequence they are reported in the header of the corresponding sequence. The order of the constituent parts can be altered using a drag and drop interface, allowing flexibility in the design of the experiment.

Additionally, the MPRAnator SNP design tool can accept a set of FASTA sequences without a list of associated SNPs and perform their incorporation into the user-designed constructs. This is particularly useful if the regulatory role of existing sequences is being investigated.

**2.3 Transmutation tool**

The Transmutation tool can be used to deconstruct a set of sequences or motifs. The software includes four options: i) scramble, ii) reverse,

iii) complement a set of motifs or sequences or iv) introduce multiple random mutations to destroy a motif or sequence’s functionality, therefore serving to generate negative controls for the MPRA SNP and Motif design tools. The transmutation tool takes a FASTA file as an input and it will output another FASTA file. The output file will either have a specific number of point mutations per sequence, or each sequence will have been scrambled through a random permutation of the nucleotides or reversed or complemented. Information regarding mutations, reversing, complementing and scrambling for each sequence is stored in the headers of the output FASTA file. Mutated nucleotides are colour-marked for visualization purposes.

**2.4 PWM Seq-Gen Tool**

The PWM Seq-Gen tool can be used to convert Positional Weight Matrices (PWMs) into k-mer sequence motifs. Each PWM must contain a header designated with “>” and 4 rows, one for each DNA nucleotide (A, C, G, T respectively). Columns in the matrix must be tab or space separated.

A set of variables allows the user to decide the type of conversion. One option is to return all k-mers of each corresponding PWM which exceed a threshold probability selected by the user. The second option is to generate probabilistic realisations of PWMs, which are represented into k-mers. If duplicates are present the user can select to remove duplicates in the output.

The output is presented in FASTA format. The header contains information regarding the probability of each k-mer occurring or the simulation number, depending on the type of convserion. The output from PWM SeqGen tool can be used as input into MPRAnator Motif Design Tool.


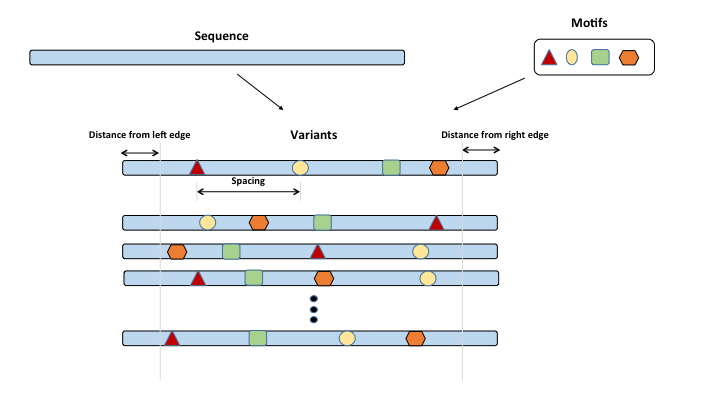


**Figure1: MPRAnator Motif design. Up to 4 motifs are substituted into each of the background sequences using all possible permutations. Distance from the edges and minimum and maximum spacing between motifs restrict the positioning of motifs in the sequences. Interval of substitution (not shown) determines the frequency of motif substitutions in consecutive sequences (substituted every X nucleotides within the inputted sequence).**


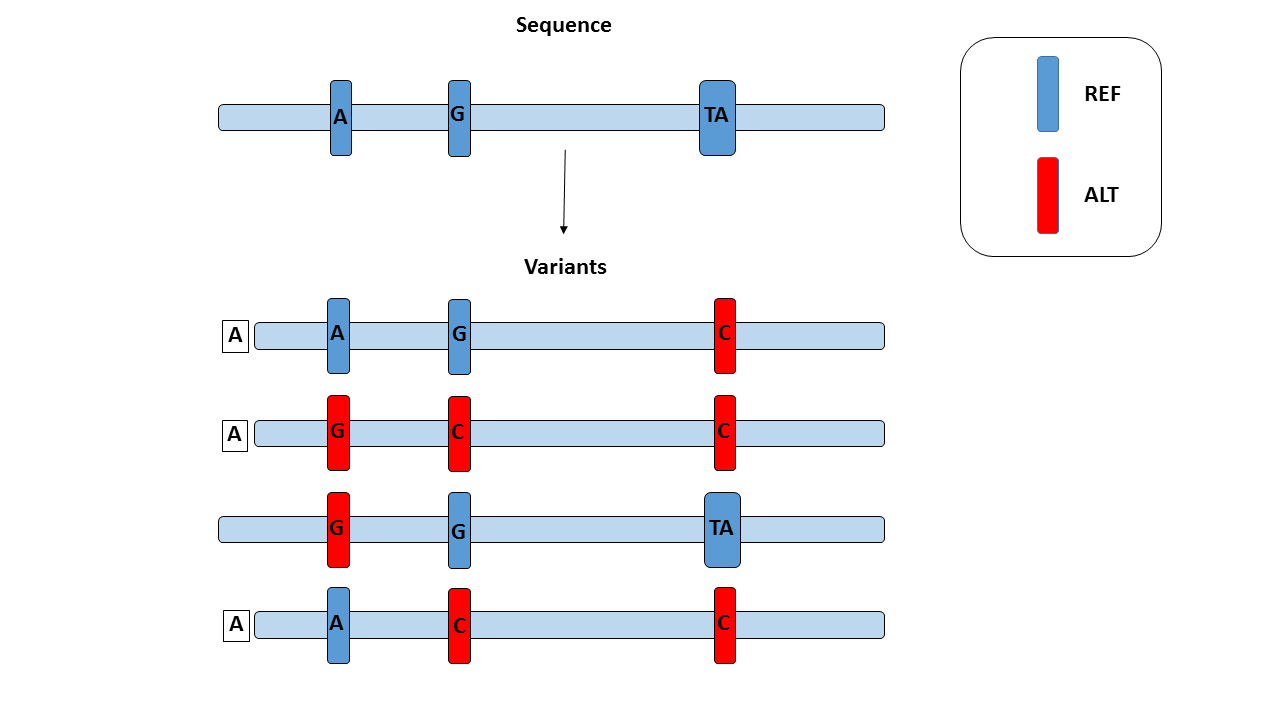


**Figure2: MPRAnator SNP design. For inserted sequences, variants are generated that contain the associated SNPs. If multiple SNPs are found in a sequence the user can select to also generate oligonucleotides with their combinations. For deletions, adenines are added at the edge of the sequence and for insertions the sequence is trimmed to maintain the same length across all output sequences.**


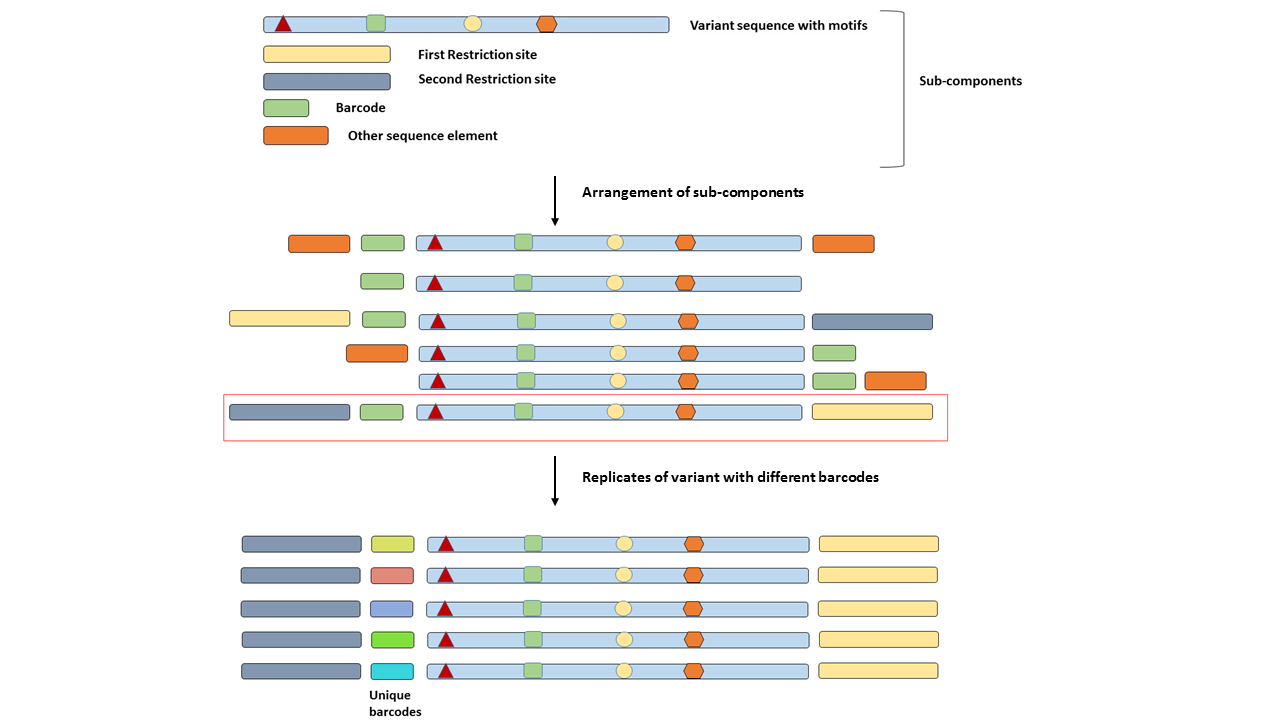


**Figure3: Modular design for final output. Sub-components can be placed in any order. Replicates of each sequence can be generated, each with distinct barcode sequence.**

**Supplementary References**

Levo,M. *et al.* (2015) Unraveling determinants of transcription factor binding outside the core binding site. *Genome Res.*, 10.1101/gr.185033.114.
